# Rosiglitazone RECORD study: glucose control outcomes at 18 months

**DOI:** 10.1111/j.1464-5491.2007.02160.x

**Published:** 2007-06-01

**Authors:** P D Home, N P Jones, S J Pocock, H Beck-Nielsen, R Gomis, M Hanefeld, M Komajda, P Curtis

**Affiliations:** Newcastle Diabetes Centre and Newcastle University UK; * GlaxoSmithKline Pharmaceuticals, Harlow, Essex UK; † Medical Statistics Unit, London School of Hygiene and Tropical Medicine London, UK; ‡ Department of Endocrinology and Metabolism Odense, Denmark; § Hospital Clinic, University of Barcelona Spain; ¶ Zentrum für Klinische Studien Forschungsbereich Endokrinologie und Stoffwechsel Dresden, Germany; ** Hôpital Pitié-Salpêtrière, Service de Cardiologie Paris, France; †† GlaxoSmithKline Pharmaceuticals, Greenford Middlesex, UK

**Keywords:** cardiovascular risk markers, HbA_1c_, RECORD, rosiglitazone combination therapy, Type 2 diabetes mellitus

## Abstract

**Aims:**

To compare glucose control over 18 months between rosiglitazone oral combination therapy and combination metformin and sulphonylurea in people with Type 2 diabetes.

**Methods:**

RECORD, a multicentre, parallel-group study of cardiovascular outcomes, enrolled people with an HbA_1c_ of 7.1–9.0% on maximum doses of metformin or sulphonylurea. If on metformin they were randomized to add-on rosiglitazone or sulphonylurea (open label) and if on sulphonylurea to rosiglitazone or metformin. HbA_1c_ was managed to ≤ 7.0% by dose titration. A prospectively defined analysis of glycaemic control on the first 1122 participants is reported here, with a primary outcome assessed against a non-inferiority margin for HbA_1c_ of 0.4%.

**Results:**

At 18 months, HbA_1c_ reduction on background metformin was similar with rosiglitazone and sulphonylurea [difference 0.07 (95% CI −0.09, 0.23)%], as was the change when rosiglitazone or metformin was added to sulphonylurea [0.06 (−0.09, 0.20)%]. At 6 months, the effect on HbA_1c_ was greater with add-on sulphonylurea, but was similar whether sulphonylurea was added to rosiglitazone or metformin. Differences in fasting plasma glucose were not statistically significant at 18 months [rosiglitazone vs. sulphonylurea −0.36 (−0.74, 0.02) mmol/l, rosiglitazone vs. metformin −0.34 (−0.73, 0.05) mmol/l]. Increased homeostasis model assessment insulin sensitivity and reduced C-reactive protein were greater with rosiglitazone than metformin or sulphonylurea (all *P* ≤ 0.001). Body weight was significantly increased with rosiglitazone compared with sulphonylurea [difference 1.2 (0.4, 2.0) kg, *P* = 0.003] and metformin [difference 4.3 (3.6, 5.1) kg, *P* < 0.001].

**Conclusions:**

In people with diabetes, rosiglitazone in combination with metformin or sulphonylurea was demonstrated to be non-inferior to the standard combination of metformin + sulphonylurea in lowering HbA_1c_ over 18 months, and produces greater improvements in C-reactive protein and basal insulin sensitivity but is also associated with greater weight gain.

## Introduction

The major therapeutic management goals for people with Type 2 diabetes are achieving optimal control of glycaemia, lipid levels and blood pressure to reduce the risk of long-term vascular complications. Treatment algorithms for the management of glycaemia have advocated a step-wise approach with diet and exercise as first-line management, followed by the addition of oral glucose-lowering agents [1]. Drug monotherapy may prove effective initially, but in the face of progressively declining islet B-cell function, the hallmark of diabetes [2,3], half the population requires combination therapy within 3 years of diagnosis [4].

The progressive nature of Type 2 diabetes, coupled with rising prevalence [5] and better adherence to HbA_1c_ targets, means that increasing numbers of people with diabetes are taking combination therapies. However, whilst clinical trials comparing glucose-lowering drugs in monotherapy are relatively common, longer-term comparisons of new dual-agent combinations with the de facto standard of metformin + sulphonylurea are rare.

Rosiglitazone Evaluated for Cardiac Outcomes and Regulation of glycaemia in Diabetes (RECORD), a 6-year, multicentre, randomized, open-label, parallel-group study of cardiovascular outcomes [6], presents an opportunity to compare rosiglitazone + metformin and rosiglitazone + sulphonylurea with metformin + sulphonylurea therapy in a large population of people with Type 2 diabetes. This paper presents a prospectively defined analysis of glucose control at 18 months in the first 1122 RECORD participants. It is the first head-to-head comparison of the two rosiglitazone-containing treatment regimens against metformin + sulphonylurea.

## Patients and methods

### Participants

**FIGURE 1:**
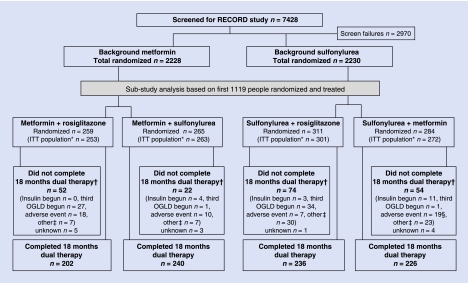
Flow diagram of the study design. This diagram includes 11 participants whose completer status was unknown at the time the database was locked for analysis, but whose status was subsequently established. *For definition of intention-to-treat (ITT) population, see text. †Includes people who moved to the Post-Randomized Treatment phase of the study, and people who withdrew completely. ‡‘Other’ reasons include lost to follow-up, consent withdrawn, sponsor terminated, non-compliance and miscellaneous reasons, including one on metformin + rosiglitazone group with ALT > 3 × upper limit of normal. §Two participants on sulfonylurea + metformin withdrew because of an adverse event in error, later resumed assigned therapy, and are not included as ‘withdrawn’. OGLD, oral glucose-lowering drug.

The RECORD protocol is described in detail elsewhere [6]. RECORD involves 330 centres in 23 countries in Europe, Australia and New Zealand. A total of 4458 individuals with Type 2 diabetes, inadequately controlled on metformin or sulphonylurea, have enrolled (Fig. 1). The current pre-specified 18-month analysis is based on data collected for the first 1122 people randomized until 15 April 2002 (598 people already on sulphonylurea; 524 already on metformin). Eligible participants had Type 2 diabetes as defined by the 1999 World Health Organization criteria [7], were aged 40–75 years, with body mass index (BMI) > 25.0 kg/m^2^, HbA_1c_ > 7.0–9.0%, and thus inadequately controlled on maximum permitted or tolerated doses of metformin or a sulphonylurea (glyburide, glimepiride or gliclazide) at study entry.

The study is conducted according to Good Clinical Practice guidelines and the Declaration of Helsinki [8]. The protocol was approved by Ethics Review Committees/Institutional Review Boards according to the local laws/customs of each participating country. Written informed consent was obtained from participants before beginning protocol-specific procedures.

### Study design

RECORD is a multicentre, randomized, open-label, comparative, parallel-group trial. Eligible participants continued to take the oral glucose-lowering drug (metformin or sulphonylurea) they were taking prior to study entry and entered a 4-week run-in period, which included reinforcement of lifestyle education, followed by randomization to additional treatment with add-on study medication. Treatment allocation, using a concealed remote telephone system, was stratified for prior glucose-lowering drug: people on a sulphonylurea randomized to either add-on rosiglitazone or metformin; those on metformin to rosiglitazone or sulphonylurea [glibenclamide (glyburide; normal/micronized), gliclazide, or glimepiride, chosen according to local practice]. Allocated drugs were non-blinded owing to the difficulties of arranging six preparations with different dosing schedules, and the impossibility of maintaining blinded allocation with asymmetric timing of insulin therapy initiation.

Throughout the study, participants were managed to target HbA_1c_ ≤ 7.0%. The randomized starting dose for rosiglitazone was 4 mg/day and those for metformin and sulphonylurea were in accordance with local clinical practice. If HbA_1c_ rose to > 7.0% after 8 weeks of randomized treatment, the dose of randomized study medication was increased towards a maximum of 4 mg rosiglitazone twice daily, 2550 mg metformin, 15 mg glyburide (or equivalent for micronized preparation), 240 mg gliclazide, or 4 mg glimepiride. If HbA_1c_ was ≥ 8.5% (confirmed by a second measurement at least 1 month later) on the maximum tolerated dose for at least 8 weeks, a third glucose-lowering agent could be added if on rosiglitazone, or insulin initiated if on metformin + sulphonylurea.

### Efficacy assessments

The primary analysis of glycaemic control was change from baseline in HbA_1c_ after 18 months’ randomized treatment. Secondary efficacy analyses included fasting plasma glucose (FPG), serum lipids, homeostasis model assessment (HOMA) basal insulin sensitivity and islet B-cell function (HOMA %B) by the equation method [9], and inflammatory/thrombotic markers [plasminogen activator inhibitor-1 (PAI-1) antigen, fibrinogen, and C-reactive protein (CRP)]. HbA_1c_, FPG and body weight were assessed at baseline and all eight follow-up visits; lipids at baseline, 6, 12 and 18 months; and other measures at baseline, 12 and 18 months. As RECORD was ongoing at the time of this 18-month analysis, safety and adverse event (AE) data including some aspects of body weight/oedema and hypoglycaemia were not analysed in order to maintain the integrity of the main study.

### Laboratory methods

A central laboratory was used for all routine laboratory assessments (Quest Diagnostics, Heston, UK). HbA_1c_ was measured by HPLC using Diabetes Control and Complications Trial (DCCT)-standardized Biorad Variant HbA_1c_ assay (Bio-Rad Laboratories, Hercules, CA, USA). FPG concentration was measured using an enzymatic method. Serum insulin (kit from Perkin Elmer, Turku, Finland) was determined by a specific two-site fluoroimmunometric assay [cross-reactivity with proinsulin: intact human proinsulin (hPI) 0.1%, des 32,33 hPI 0.4%, des 64,65 hPI 66%]. Intact proinsulin was measured using a two-site fluoroimmunometric assay (typically < 1% cross-reaction with insulin and 32–33 split proinsulin at 2500 pmol/l and 400 pmol/l, respectively; no detectable cross-reaction with C-peptide]. Total and high-density lipoprotein (HDL) cholesterol were analysed by Olympus cholesterol esterase assays (Olympus UK, Southall, UK), and low-density lipoprotein (LDL) cholesterol was calculated using the Friedewald equation. Triglycerides were measured by enzymatic determination of glycerol using Olympus reagents. Non-esterified fatty acids (NEFA) were measured by enzymatic colorimetric assay (Wako Chemicals, Neuss, Germany). PAI-1 antigen was quantified using a Biopool TintElize (Ventura, CA, USA) enzyme immunoassay kit. Highly sensitive CRP was measured by fixed-time nephelometry (Dade Behring, Milton Keynes, UK) and fibrinogen by photo-optical clot detection in plasma on adding thrombin.

### Statistical analysis

The primary objective was to test whether the 18-month mean change from baseline HbA_1c_ for the intention-to-treat (ITT) population (all randomized, treated and with at least one data point post-randomization) with rosiglitazone oral combination therapy was at least as good as the respective controls receiving metformin + sulphonylurea. The non-inferiority criterion (upper bound 95% CI of difference) was preset at 0.4 % units. With 260 participants per arm and change from baseline sd of 1.4% (actual ∼1.0%), the study was estimated to have 90% power.

The primary ITT analysis of treatment difference at 18 months was by repeated-measures analysis (based on all available data) using a model including terms for treatment and baseline by visit interaction, using an unstructured covariance matrix to model the within-patient variability for each treatment group. A supportive per-protocol analysis excluding participants with prospectively defined major protocol deviations at baseline (principally regarding background glucose-lowering treatment and HbA_1c_) or during the 18-month treatment period (use of prohibited medication, non-compliance with the treatment algorithm) was also performed. Participants who stopped dual-combination therapy, including those who progressed to triple-combination therapy or started insulin, had their data censored from the time dual-combination therapy was stopped. Analyses of FPG and body weight data used the same methodology as for HbA_1c_.

A responder analysis, including the proportion of participants with a reduction in HbA_1c_ ≥ 0.7% and the proportion achieving a predefined target of ≤ 7.0%, both at 18 months, compared treatment groups using logistic regression modelling with treatment and baseline HbA_1c_ as factors. Conservatively, people with missing data at 18 months were taken as non-responders.

For fasting insulin, proinsulin, proinsulin:insulin ratio, HOMA estimates of insulin sensitivity and islet B-cell function, and for inflammatory/thrombotic markers, summary statistics using log-transformed data were produced. Between-group differences within each stratum were calculated at 18 months for insulin sensitivity and inflammatory/thrombotic markers using ancova on observed case data at 18 months, adjusted for baseline value. This methodology was also used for analyses of lipid parameters using untransformed data.

All significance tests and confidence intervals were two-sided and performed or constructed at the 5% significance level. Analyses were conducted using SAS for Windows (version 8.2; SAS Institute, Cary, NC, USA).

## Results

### Baseline characteristics and withdrawals

Baseline characteristics were well matched between randomized groups within the background metformin and background sulphonylurea strata (Table 1). However, participants in the background metformin stratum had lower age, higher BMI and a shorter duration since diagnosis than those in the background sulphonylurea stratum.

**Table 1:** Clinical and baseline characteristics of the population studied

	Background metformin		Background sulphonylurea	
		
	Rosiglitazone	Sulphonylurea	Rosiglitazone	Metformin
Patients (*n*)	259	265	311	284
Age (years)	57 ± 8	57 ± 8	61 ± 8	60 ± 8
Male [*n* (%)]	141 (54)	139 (52)	152 (49)	149 (52)
Europid [*n* (%)]	257 (99)	263 (99)	309 (99)	281 (99)
Time from diagnosis (years)	6.1 ± 4.3	7.0 ± 5.6	7.9 ± 5.7	8.1 ± 5.4
Body weight (kg)	93 ± 17	91 ± 15	84 ± 14	83 ± 12
Body mass index (kg/m^2^)	32.7 ± 5.4	32.3 ± 4.8	30.1 ± 3.8	29.8 ± 3.9
HbA_1c_ (%)	7.9 ± 0.70	7.8 ± 0.66	8.0 ± 0.69	8.0 ± 0.77
Fasting plasma glucose (mmol/l)	9.7 ± 2.38	9.7 ± 2.23	10.2 ± 2.54	10.1 ± 2.24
Homeostasis model assessment %B (%)	30.6 (19.5, 44.2)	30.9 (17.6, 47.3)	24.3 (15.9, 37.9)	22.2 (14.2, 36.8)
Homeostasis model assessment %S (%)	29 (19, 43)	30 (17, 45)	29 (20, 45)	32 (21, 49)
C-reactive protein (mg/l)	3.6 (1.7, 7.2)	3.0 (1.4, 7.1)	2.5 (1.3, 4.6)	2.9 (1.2, 5.2)
Plasminogen activator inhibitor-1 antigen (µg/l)	60 (39, 84)	55 (38, 87)	60 (40, 91)	62 (40, 92)
Fibrinogen (g/l)	3.2 (2.8, 3.7)	3.2 (2.7, 3.6)	3.2 (2.8, 3.7)	3.3 (2.9, 3.8)
Insulin (pmol/l)	61 (41, 89)	57 (39, 97)	57 (36, 80)	53 (34, 71)
Proinsulin (pmol/l)	9.1 (5.8, 16.0)	9.6 (6.3, 15.7)	11.8 (7.7, 19.6)	10.9 (7.0, 17.5)
Proinsulin:insulin ratio (pmol/pmol)	0.16 (0.10, 0.25)	0.16 (0.10, 0.25)	0.23 (0.14, 0.36)	0.23 (0.15, 0.36)
Total cholesterol (mmol/l)	5.4 ± 0.92	5.4 ± 1.02	5.6 ± 1.04	5.6 ± 1.09
Triglycerides (mmol/l)	2.2 ± 1.11	2.1 ± 1.32	2.1 ± 1.88	2.1 ± 1.45
High-density lipoprotein cholesterol (mmol/l)	1.2 ± 0.27	1.2 ± 0.28	1.2 ± 0.31	1.2 ± 0.32
Low-density lipoprotein cholesterol (mmol/l)	3.2 ± 0.79	3.2 ± 0.84	3.5 ± 0.87	3.5 ± 0.96
Non-esterified fatty acids (mmol/l)	0.63 ± 0.22	0.63 ± 0.24	0.66 ± 0.25	0.64 ± 0.24

Number (%), mean ± sd, or median (IQR).

All but three participants began their randomized medication; 202 were withdrawn before 18 months. More participants in the metformin + rosiglitazone group (20%) ceased dual-combination therapy than in the metformin + sulphonylurea group (8%; Fig. 1). More participants in the metformin + rosiglitazone group progressed to triple therapy (10%) than those to insulin in the metformin + sulphonylurea group (2%). Similar proportions of participants in the sulphonylurea + rosiglitazone and sulphonylurea + metformin groups ceased dual-combination treatment (24 and 19%, respectively), but more patients in the sulphonylurea + rosiglitazone group progressed to triple therapy (11%) than those to insulin in the sulphonylurea + metformin group (4%), while more people in the sulphonylurea + metformin group (7%) than the sulphonylurea + rosiglitazone group (1%) stopped randomized treatment as a result of an AE (Fig. 1).

### Glucose control end points

At 18 months, the reductions in HbA_1c_ in the rosiglitazone groups were similar to those achieved in the respective control groups (Table 2). In both strata, the upper limit of the 95% CI for the treatment difference was 0.2%, half the criterion for non-inferiority. The results of the per-protocol analysis supported the ITT analysis (data not shown).

**Table 2:** Change from baseline at 18 months in measures of glucose control, insulin sensitivity, body weight, inflammatory/thrombotic markers and lipid measures

	Background metformin			Background sulphonylurea		
	Background metformin			Background sulphonylurea		
	Rosiglitazone	vs.	Sulphonylurea	Rosiglitazone	vs.	Metformin
ITT population (*n*)	253		263	301		272
Completers (*n*)	202		240	236		226
HbA_1c_ (%)						
Change	−0.48 (−0.59, −0.36)		−0.55 (−0.66, −0.44)	−0.55 (−0.67, −0.44)		−0.61 (−0.70, −0.51)
Difference, *P*		0.07 (−0.09, 0.23), NS			0.06 (−0.09, 0.20), NS	
Fasting plasma glucose (mmol/l)						
Change	−1.5 (−1.78, −1.29)		−1.2 (−1.46, −0.89)	−2.0 (−2.26, −1.66)		−1.6 (−1.87, −1.36)
Difference, *P*		−0.36 (−0.74, 0.02), 0.062			−0.34 (−0.73, 0.05), 0.089	
Homeostasis model assessment %B (% baseline)						
Change	13.4 (4.2, 23.3)		38.8 (28.3, 50.1)	33.0 (22.2, 44.7)		25.4 (15.1, 36.6)
Difference, *P*		−18.3 (−27.2, −8.4), < 0.001			6.1 (−6.0, 19.7), NS	
Homeostasis model assessment %S (% baseline)						
Change	56 (42, 70)		11 (2, 20)	65 (53, 77)		36 (26, 46)
Difference, *P*		40 (25, 58), < 0.001			21 (10, 34), < 0.001	
Body weight (kg)						
Change	2.3 (1.7, 2.9)		1.1 (0.6, 1.6)	3.4 (2.9, 4.0)		−0.9 (−1.4, −0.4)
Difference, *P*		1.2 (0.4, 2.0), 0.003			4.3 (3.6, 5.1), < 0.001	
C-reactive protein (% baseline)						
Change	−41 (−48, −33)		−6 (−17, 6)	−36 (−43, −28)		−16 (−26, −6)
Difference, *P*		−37 (−47, −24), < 0.001			−24 (−35, −10), 0.001	
Plasminogen activator inhibitor-1 antigen (% baseline)						
Change	−5.7 (−13.9, 3.3)		7.0 (−1.7, 16.4)	−11.1 (−18.5, −3.1)		−11.2 (−18.7, −3.0)
Difference, *P*		−12 (−22, −0.2), 0.047			0 (−12, 13), NS	
Fibrinogen (% baseline)						
Change	2.1 (−0.9, 5.1)		10.9 (7.9, 14.0)	3.2 (0.9, 5.7)		7.3 (4.7, 9.9)
Difference, *P*		−8.0 (−11.6, −4.2), 0.001			−3.8 (−7.0, −0.5), 0.024	
Total cholesterol (mmol/l)						
Change	0.37 (0.23, 0.51)		−0.16 (−0.29, −0.03)	0.46 (0.33, 0.59)		−0.10 (−0.24, 0.04)
Difference, *P*		0.53 (0.34, 0.71), < 0.001			0.56 (0.36, 0.75), 0.001	
Triglycerides (mmol/l)						
Change	0.40 (0.25, 0.55)		0.15 (0.01, 0.29)	0.24 (0.06, 0.42)		0.17 (−0.02, 0.36)
Difference, *P*		0.26 (0.05, 0.47), 0.016			0.06 (−0.20, 0.32), NS	
High-density lipoprotein cholesterol (mmol/l)						
Change	0.08 (0.05, 0.11)		0.02 (−0.01, 0.05)	0.10 (0.07, 0.13)		0.08 (0.05, 0.11)
Difference, *P*		0.06 (0.02, 0.10), 0.001			0.01 (−0.02, 0.05), NS	
Low-density lipoprotein cholesterol (mmol/l)						
Change	0.04 (−0.07, 0.15)		−0.26 (−0.36, −0.16)	0.19 (0.08, 0.30)		−0.29 (−0.40, −0.18)
Difference, *P*		0.30 (0.16, 0.45), 0.000			0.48 (0.32, 0.64), 0.000	
Non-esterified fatty acids (mmol/l)						
Change	−0.07 (−0.10, −0.04)		0.04 (0.01, 0.07)	−0.06 (−0.10, −0.02)		0.06 (0.02, 0.10)
Difference, *P*		−0.11 (−0.15, −0.06), 0.000			−0.12 (−0.17, −0.07), 0.000	

Data are model-adjusted mean (95% CI), or number.

**FIGURE 2:**
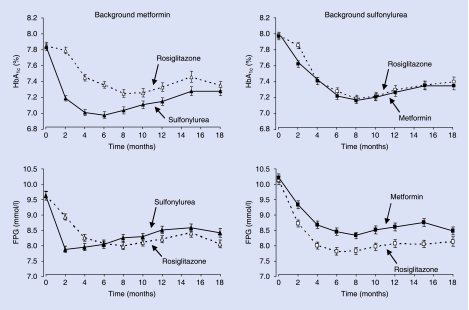
Time course for HbA_1c_ (upper panels) and fasting plasma glucose (FPG; lower panels) during the 18-month treatment period. Data are model adjusted mean ± se. Left-hand panels show addition of rosiglitazone (▵; *n* = 253) or sulfonylurea (▴; *n* = 263) to metformin; right-hand panels show addition of rosiglitazone (□; *n* = 301) or metformin (▪; *n* = 272) to sulfonylurea.

In the background metformin stratum, the HbA_1c_ response was more rapid with sulphonylurea than rosiglitazone (Fig. 2), with a significant mean adjusted treatment difference (*P* < 0.001) in favour of metformin + sulphonylurea at 6 months [difference 0.37 (95% CI 0.25, 0.49)%]. However, this superiority was not sustained beyond 8–12 months (Fig. 2) and, at 18 months, the mean adjusted treatment difference was no longer significant between the two groups (Table 2). In the background sulphonylurea stratum, the trajectory of HbA_1c_ reduction was similar for rosiglitazone and metformin groups (Fig. 2), with no significant differences at 6 or 18 months.

A reduction in HbA_1c_ ≥ 0.7% from baseline was achieved at 18 months in 35 and 45% of the background metformin participants on rosiglitazone and sulphonylurea, respectively [odds ratio (OR) 0.62 (95% CI 0.42, 0.90), *P* = 0.012] and 45 and 37% of background sulphonylurea participants using rosiglitazone and metformin [OR 1.47 (1.02, 2.10), *P* = 0.037]. An HbA_1c_ ≤ 7.0% at 18 months was achieved by 35 and 39% of participants comparing rosiglitazone with sulphonylurea, and 37 and 31% comparing rosiglitazone with metformin (both comparisons NS).

At 18 months, the apparently greater reductions in FPG in the rosiglitazone groups did not reach statistical significance (Table 2). On background metformin, FPG fell rapidly after initiating a sulphonylurea (within 2 months) but this advantage was lost by 6 months (Fig. 2). In both rosiglitazone groups, initial FPG reduction was slower, steadying at 6–8 months. Metformin + sulphonylurea showed a similar trajectory to the rosiglitazone groups (Fig. 2).

### Insulin sensitivity and islet B-cell function

In both background treatment strata, 18-month HOMA-estimated basal insulin sensitivity was substantially increased in the rosiglitazone groups compared with the respective controls (both *P* < 0.001; Table 2). The effect of metformin on insulin sensitivity was about half that of rosiglitazone. Both rosiglitazone and sulphonylurea when added to metformin increased HOMA %B, but this increase was greater with sulphonylurea (*P* < 0.001; Table 2). Rosiglitazone or metformin added to background sulphonylurea also increased HOMA %B, and to a similar extent (Table 2).

At 18 months in both strata, rosiglitazone-treated patients had greater mean reductions in fasting plasma insulin than the respective controls [metformin strata: rosiglitazone −11.1 (−17.5, −4.7) vs. sulphonylurea 4.4 (−1.5, 10.3) pmol/l; sulphonylurea strata: rosiglitazone −15.4 (−19.8, −11.1) vs. metformin −5.9 (−9.4, −2.5) pmol/l]. Similar differences were obtained for proinsulin for rosiglitazone vs. sulphonylurea [−4.8 (−6.0, −3.5) vs. 1.8 (0.4, 3.1) pmol/l], but with overlap for the rosiglitazone vs. metformin groups [−6.4 (−9.2, −3.7) vs. −3.5 (−4.7, −2.3) pmol/l]. Rosiglitazone resulted in a greater reduction in proinsulin:insulin ratio than sulphonylurea [−22.3 (−28.9, −15.1) vs. 0.9 (−6.6, 9.1)%], whereas similar decreases were observed when rosiglitazone was contrasted with metformin [−15.0 (−21.3, −8.2) vs. −17.1 (−23.5, −10.2)%].

### Body weight

Increases in body weight were observed in both arms of the metformin stratum; however, this increase was greater with rosiglitazone than sulphonylurea (*P* = 0.003; Table 2). In the sulphonylurea stratum there was a significant increase in body weight with rosiglitazone compared with a slight decrease with metformin (*P* < 0.001).

### Lipids and inflammatory/thrombotic markers

In both background treatment strata, rosiglitazone increased total cholesterol and LDL cholesterol and reduced NEFA at 18 months compared with control groups (Table 2). An increase in HDL cholesterol and triglycerides was observed with rosiglitazone compared with sulphonylurea (0.08 vs. 0.02 mmol/l, *P* = 0.001; 0.40 vs. 0.15 mmol/l, *P* = 0.016, respectively), but not with metformin (Table 2).

At 18 months, PAI-1 antigen decreased from baseline with rosiglitazone, with a significant difference compared with sulphonylurea (−5.7 vs. 7.0%, *P* = 0.047; Table 2); rosiglitazone and metformin did not differ (Table 2). In both rosiglitazone groups, there were statistically significant reductions in CRP compared with respective controls (Table 2), even against metformin + sulphonylurea which showed a small fall from baseline. The level of fibrinogen increased in all treatment groups, but the magnitude of the increase was significantly smaller in the rosiglitazone groups compared with controls, with the greatest contrast against sulphonylurea (Table 2).

## Discussion

This pre-defined sub-study of RECORD has the strength of being the first randomized clinical trial to compare add-on rosiglitazone combination therapy with both sulphonylureas and metformin against the standard combination of metformin + sulphonylurea, across a wide range of surrogate outcomes. Other strengths of the study include its size and longer-term duration, together with a wide population base. Weaknesses include the open-label design and the exclusion of clinically significant data, including AEs, reporting of which might affect the integrity of the underlying 6-year study. Similar data for people with poor blood glucose control have recently been published for pioglitazone [10]. Importantly, average blood glucose control at entry to the current study on metformin or sulphonylurea therapy was not too poor, and the dose of study medication was not fixed or force-titrated, but adjusted to achieve target HbA_1c_ ≤ 7.0%. This was in line with some guidelines at the time of study design, and is such that the glucose control achieved, if not optimal, appears not untypical of current clinical practice.

The primary outcome findings from the study demonstrate that following 18 months’ treatment a similar degree of overall glucose-lowering efficacy is achieved with add-on rosiglitazone as that achieved with add-on metformin and sulphonylurea. As the improvement of HbA_1c_ in the two arms was the same with rosiglitazone, the study also suggests that metformin and sulphonylureas have similar glucose-lowering efficacy after 1.5 years. None of these observations excludes the possibility that there are sub-populations or individuals in whom the efficacy of the drugs will differ.

However, the rate of change of glucose-lowering efficacy does differ among the three drugs, the data presented being consistent with previous studies [10]. Thus, the sulphonylureas have a rapid onset of action and effect on direct blood glucose measurements (FPG in Fig. 2), but a rate of fall of HbA_1c_ which, allowing for kinetics of glycated haemoglobin turnover, suggests nearly instantaneous effects on blood glucose control. Rosiglitazone, as pioglitazone, has a much slower onset of effect; this is also true, although not generally appreciated in clinical practice, of metformin (Fig. 2). It seems reasonable to suggest that the effects of sulphonylureas in stimulating insulin secretion are nearly instantaneous, but that restoration of insulin sensitivity with the thiazolidinediones takes time.

The effect of sulphonylureas, however, is not sustained, the data here being consistent with studies showing improvement then waning of islet B-cell function over the first 10 months’ therapy [10]. Accordingly, despite the improvement in blood glucose control persisting to 18 months, and presumably still reflecting improved pancreatic function [11], proinsulin and proinsulin:insulin were unchanged with sulphonylureas, but improved with rosiglitazone and metformin. In the longer term (3–6 years), metformin and sulphonylureas are associated with similar rates of loss of islet B-cell function [11], but the more rapid loss of function with sulphonylureas after the initial gain is not understood. Long-term data for rosiglitazone will be an outcome of the RECORD study at its 6-year termination, and have recently become available from the ADOPT study for people with lesser degrees of impaired glucose metabolism [12].

Reductions in FPG observed with rosiglitazone were generally greater than would be predicted from the change in HbA_1c_. This might be explained by a greater effect on postprandial glycaemia by metformin and/or sulphonylurea, but this hypothesis cannot be tested within this study as no postprandial measurements were made. The extent of the HbA_1c_ reduction at 18 months (0.5–0.6%), from baseline levels of 7.8–8.0%, explains the small proportion of participants reaching the conservative glucose control target by 18 months. If current guideline targets from International Diabetes Federation (IDF) and American College of Endocrinologists (ACE)/American Association of Clinical Endocrinologists (AACE) are to be met [1,13], this emphasizes that combination therapy will need to be initiated early in the clinical history of most people with diabetes and measures to achieve this, such as guideline implementation initiatives and combination tablets, need further study. In the present study, despite the similar mean 18-month HbA_1c_, differences were observed in responder rates, with more rosiglitazone than metformin participants achieving a reduction of ≥ 0.7% in the sulphonylurea stratum, while fewer so responded on rosiglitazone than sulphonylurea in the metformin stratum. The apparent inconsistency with mean HbA_1c_ may in part be explained by some non-normality in all groups, and the conservative assumptions made about those with missing data in the responder analysis. The non-normality may have arisen from there being subgroups of people who respond better to one drug or the other, but confirmation of this by other large trials would be needed.

The number of participants moving off dual-combination treatment was higher for the rosiglitazone groups than the respective control groups. This was particularly noticeable for the sulphonylurea comparison, and may reflect the early and rapid effect of these drugs noted above, particularly as the entry criteria for HbA_1c_ (< 9.0%) were above the threshold for discontinuation (8.5%). Additionally, this was an open asymmetric study [6], where the comparator groups move to insulin while the rosiglitazone group has another oral agent added. This is likely to have inhibited the comparator groups from ceasing oral combination therapy [14,15].

The improvements observed with rosiglitazone in insulin sensitivity have previously been reported in other smaller, shorter studies [16–19], and with pioglitazone. Interestingly, the effect of metformin was much lower, even though this drug is sometimes termed an insulin sensitizer, implying that the effect of metformin on glucose-lowering involves mechanisms other than improving hepatic performance in response to insulin. There was no evidence of a secondary effect of sulphonylureas on basal insulin sensitivity. These improvements in insulin sensitivity were observed in the face of substantial weight gain with rosiglitazone. This apparent paradox is a well-known feature of thiazolidinedione pharmacology and may be related to preferential deposition of subcutaneous and not visceral fat [18,20]. It is known from the UK Prospective Diabetes Study (UKPDS) that metformin improves HOMA estimates of islet B-cell function, although to a much smaller degree than sulphonylureas, but the mechanism (reduction in glucose toxicity or reduction in demand on the islet B-cell) is not known. A similar change is shown here for rosiglitazone.

Although there were no restrictions on the initiation or continued use of lipid-lowering therapy in RECORD, this does not appear to have substantially confounded the assessment of 18-month lipid end points, as they confirmed the previously reported effects of rosiglitazone in increasing total, LDL and HDL cholesterol [21]. The significance of the changes in LDL and thus total cholesterol remain difficult to interpret after use of thiazolidinediones, because the nature of the LDL lipoprotein particles is markedly changed by these drugs to a less-dense phenotype [22].

The improvements in CRP, PAI-1 antigen, and fibrinogen observed with rosiglitazone combination therapy are also consistent with previous monotherapy data [23,24], and are shown to be sustained. Metformin again affected CRP, albeit much less than rosiglitazone; both had the same effect on PAI-1. Sulphonylureas were not active in these respects, or possibly (for PAI-1) worsened this prothrombotic marker. Given that these three measures are risk markers for cardiovascular (CV) events in people with Type 2 diabetes [25–27], and given that metformin appears to prevent CV events beyond what can be predicted by its glucose-lowering effect, the results of the CV outcomes phase of RECORD will be awaited with interest, particularly in the light of the findings in the PROspective PioglitAzone Clinical Trial In MacroVascular Events (PROactive) study [28].

In conclusion, after 18 months’ treatment, rosiglitazone in combination with metformin or sulphonylurea, is as effective in lowering HbA_1c_ in people with Type 2 diabetes as the standard combination of metformin + sulphonylurea, and produces greater improvements in CRP and insulin sensitivity but is also associated with greater weight gain.

## Competing interests

The RECORD study sponsor is GlaxoSmithKline, the manufacturer of rosiglitazone. Members of the Steering Committee, Data Safety and Monitoring Board, and Clinical Endpoints Committee, or their institutions, are remunerated for time and expenses in connection with this and other activities relating to the company and product. Local investigators and/or their institutions are paid fees per participant for study activities; some have other relationships with the company. PDH, SJP, HBN, RG, MH, and MK are members of the RECORD steering committee. PC and NPJ are employees of GlaxoSmithKline.

## Abbreviations

AE: adverse event
BMI: body mass index
CRP: C-reactive protein
CV: cardiovascular
FPG: fasting plasma glucose
HDL: high-density lipoprotein
HOMA: homeostasis model assessment
hPI: intact human proinsulin
ITT: intention to treat
LDL: low-density lipoprotein
NEFA: non-esterified fatty acids
PAI-1: plasminogen activator inhibitor-1
RECORD: Rosiglitazone Evaluated for Cardiac Outcomes and Regulation of glycaemia in Diabetes
UKPDS: UK Prospective Diabetes Study
